# A Retrospective Analysis of the Effects of Cardiac Rehabilitation on Health Markers and Performance Outcomes among Diabetic and Non-Diabetic Patients with Coronary Artery Bypass Grafting and Percutaneous Coronary Intervention

**DOI:** 10.3390/sports12050122

**Published:** 2024-04-28

**Authors:** Amy N. McKeever, Phillip C. Drouet, Jesus A. Vera, William E. Thomas, Jared W. Coburn, Pablo B. Costa

**Affiliations:** 1 Cardiac Rehabilitation Program, Providence St. Jude Medical Center, Fullerton, CA 92835, USA; 2 Department of Kinesiology, California State University, Fullerton, CA 92831, USA

**Keywords:** blood glucose, cardiovascular disease, intervention, lipids, phase 2

## Abstract

Background: The aim of this study was to investigate the effects of cardiac rehabilitation on health markers and performance outcomes among diabetic and nondiabetic patients with coronary artery bypass grafting (CABG) and percutaneous coronary intervention (PCI). Methods: One hundred and ninety-seven patients with PCI and CABG, who attended phase 2 cardiac rehabilitation, were included in the study. Patient data were separated by cardiac diagnosis, (PCI and CABG), diabetes category (diabetic and nondiabetic), number of sessions attended (12–24 or 25–36), and time (pre- to post-test). The Duke Activity Score Index and Patient Health Questionnaire-9 questionnaires and measurements for total cholesterol, high-density lipoprotein, low-density lipoprotein, triglycerides, and, if diabetic, A1c and fasting blood glucose, were taken at baseline and upon completion of the program. Results: High-density lipoprotein (*p* < 0.001), diastolic blood pressure (*p* = 0.004), Duke Activity Score Index questionnaire (*p* < 0.001), Patient Health Questionnaire-9 (*p* < 0.001), and A1c (*p* = 0.003) significantly improved from pre- to post-testing. Total cholesterol (*p* < 0.001) and low-density lipoprotein (*p* < 0.001) for the 25–36 nondiabetic PCI group significantly decreased. Triglycerides decreased for all 12–24 session groups (*p* = 0.015). Fasting blood glucose significantly decreased (*p* = 0.037) for the 12–24 PCI group with diabetes. No significant interactions were found for systolic blood pressure and body weight. Conclusion: Cardiac rehabilitation resulted in significant improvements in the lipid panel, diastolic blood pressure, and questionnaire results, regardless of the number of sessions attended. However, no significant benefits for systolic blood pressure were observed.

## 1. Introduction

Cardiovascular disease (CVD) is the leading cause of mortality throughout the world [1]. In the United States, CVD accounted for 931,578 deaths in 2021 [1]. The incidence of CVD has continued to rise throughout the years, as avoidable risk factors such as type 2 diabetes and obesity have been on the rise [2]. Early action, intervention, and prevention for modifiable risk factors for developing CVD have been classified as necessary and crucial [3].

Risk factors that are unavoidable are age, men over 45 years of age, and women over 55, in addition to family history, such as those who have had a direct genetic relationship with someone who has had a cardiac event or bypass surgery [4]. Other risk factors include cigarette smoking, a sedentary lifestyle, obesity, hypertension, dyslipidemia, or being pre-diabetic [4]. Because prevalence is common, those that have undergone a cardiac event are recommended to participate in primary and secondary prevention [5]. 

Phase 2 cardiac rehabilitation (CR) is widely recognized as a secondary prevention method and class one recommendation for recovery after a cardiac event, surgery, or stable cardiac condition [5]. Outpatient CR involves medical evaluation, psychosocial assessment, behavioral intervention, exercise prescription, education, cardiac risk factor modifications, and medication and lifestyle management, which are designed to improve heart health and functional status [5,6]. Consisting of 36 sessions, patients undergo three months of a supervised multidisciplinary regimen that begins outside of the hospital through referral or personal choice [5]. Programs also include exercise sessions that are purposely designed to monitor patients and their response to exercise through observing symptoms such as heart rhythm, heart rate, and blood pressure [6].

CR programs are tailored to decrease rates of morbidity and mortality, and to alleviate symptoms such as angina, dyspnea, and exercise tolerance [6]. It was found that those who had participated in an exercise-based CR program had lower risks of cardiac mortality [7,8] and all-cause mortality [7,8] at long-term follow up by 26%, a lower risk of reinfarction by 18%, and a 23% decreased risk of overall hospitalization [8]. In addition, patients were also found to benefit from improved physical health, quality of life, psychosocial support, and general health [8]. Programs consist of in-person attendance for two to three sessions each week, for three to four months [6]. Therefore, the benefits of phase 2 CR include physical and emotional improvements combined with a decreased risk in mortality [5,9]. 

Percutaneous coronary intervention (PCI), and coronary artery bypass grafting (CABG) are both common diagnoses in CR [4]. PCI and CABG are both procedures performed to open or replace occluded arteries that are no longer functional for proper blood supply [4]. Both procedures are often followed with phase 2 CR to manage any symptoms and help educate the patient on lifestyle changes to prevent reoccurrence [6]. In addition, these three diagnoses are often accompanied by type 1 or type 2 diabetes mellitus [10]. With a diabetes diagnosis, the risk for a cardiovascular event is two to four times more likely than without, due to multiple comorbidities such as obesity, insulin resistance, and high blood pressure, which is a major risk factor for stroke and plaque development [4,10,11]. Patients also commonly exhibit high levels of low-density lipoprotein (LDL), and low levels of high-density lipoprotein (HDL) combined with high amounts of triglycerides [11]. Therefore, because of a diabetes diagnosis, health markers such as blood glucose, blood pressure, cholesterol, and body mass may be influenced by insulin resistance or insulin deficiency [11]. 

When comparatively examining diabetic and nondiabetic patients, the role of regular CR training following a cardiac event, the number of sessions attended, diagnosis, and health markers have yet to be comprehensively studied altogether. There have been significant findings for studies that isolate these variables, but the gap in the literature remains. For example, a study conducted by Medicare found that those (*n* = 30,000) who attended the full three months of prescribed CR had a 47% lower mortality rate than those who only came to one session [12]. For every six additional sessions, a 6% reduction in mortality was correlated [12]. In addition to a reduction in mortality, CR has been shown to improve health markers in patients with CVD and diabetes due to frequent exercise [13]. Research has shown that consistent and frequent exercise implementation [4] leads to improvements in total cholesterol [14], high-density and low-density lipoprotein [14], blood pressure [15], mental health [16], blood glucose [17], metabolic equivalent [18], and body mass [19], all of which are commonly observed through a patient’s blood panel and questionnaires given initially at CR and upon completion [20]. 

There are appropriate ranges for each health outcome being observed. For cholesterol, total cholesterol should be no more than 200 mg/dL, triglycerides should be below 150 mg/dL, high-density lipoprotein should be near 40 mg/dL for men and 50 mg/dL for women, not exceeding 60 mg/dL, and low-density lipoprotein should be less than 130 mg/dL [21]. In order for blood pressure to fall within the normal range, systolic blood pressure should be below 120 and diastolic blood pressure below 80 [21]. Consistent readings higher than this at rest may indicate a patient has elevated blood pressure or is hypertensive [21]. Fasting blood glucose should be less than or equal to 100 mg/dL and A1c should be below 5.7% [21].

Thus, for patients with PCI and CABG combined with glycemic control conditions, health markers on performance outcomes were calculated comparatively and under the influence of the number of CR sessions completed. Therefore, the purpose of this study was to compare the effects of CR on health markers between glycemic control conditions (diabetic and nondiabetic patients), diagnosis (CABG and PCI), and the number of sessions completed (twelve to twenty-four or twenty-five to thirty-six). The secondary purpose was to isolate the health markers, regardless of diagnosis, and compare the values from intake to discharge, examining for any significant interactions. It was hypothesized that, regardless of diagnosis, those who attended more CR sessions would have a greater improvement in all health markers. Secondarily, questionnaires given to patients would show improvement among both groups, regardless of the amount of CR sessions attended.

## 2. Methods

### 2.1. Subjects

A total of 197 patients (158 men and 39 women) between ages 36 and 96 (mean ± SD, 68.74 ± 11.13) who attended outpatient CR at a medical center from January 2018 to December 2021 were included in the study. Table 1 displays the breakdown of patient characteristics. Patients had a primary cardiac diagnosis of either CABG or PCI. Other inclusion criteria were attendance of twelve or more CR sessions and completion of intake and discharge blood work, the Patient Health Questionnaire-9 (PHQ-9) [22], and Duke Activity Status Index (DASI) questionnaire [23]. If a patient had been in the CR program more than once within these time frames, only their most recent cardiac event was included in the study. All patients were on some form of cardioprotective medication, which included but was not limited to aspirin, beta blockers, angiotensin converting enzyme inhibitors, angiotensin receptor blockers, and statins. Recommendations for the structure of this CR program were developed by the American College of Sports Medicine [21] and the American Association of Cardiac and Pulmonary Rehabilitation [24]. This study was deemed exempt by the university’s (HSR-22-23-333) and hospital’s (STUDY2023000209) Institutional Review Boards.

### 2.2. Measurements

Blood panels and questionnaires were requested from the patient following their cardiac event, but prior to attending the first session and ten weeks into the program. If a patient did not complete the entire program, values were requested two weeks before their last session. The blood panel included the values for total cholesterol, HDL, LDL, triglycerides and, if diabetic, fasting blood glucose and A1C. The PHQ-9 [22] and the DASI questionnaires [23] were administered. The PHQ-9 was used to observe the level of depression of the patient and the DASI was used to assess the level of physical activity of the patient. Systolic blood pressure, diastolic blood pressure, and body mass (kg) measurements were collected at the first CR session and last CR session. All initial and ending measurements were entered into a secure database.

### 2.3. Exercise Program

At the first session, patients received an initial evaluation, which included a review of health history. An individualized supervised exercise program was assigned and determined based on the patient’s cardiac diagnosis, sternotomy versus PCI, estimated resting oxygen consumption from the DASI, age, time since their cardiac event, medications, history of previous engagement in exercise, medical history, orthopedic limitation, hemodynamic response to exercise, anginal threshold, level of dyspnea, personal goals, and their glycemic response to exercise if patients were diabetic. Patient’s metabolic equivalent was assessed by the DASI questionnaire [23] and then used for determining the levels and intensities of each exercise in their exercise prescription. With all of these factors taken into consideration, the nurses and exercise physiologists created an individualized treatment plan for each patient.

Thirty-six sessions were offered to each patient. This required each patient to attend CR three times a week, for one hour, for the duration of three to four months. Patients were scheduled to attend each session in that order, unless a conflict arose and they canceled. Conflicts included, but were not limited to, sickness, family matters, and lack of transportation. The hour-long session consisted of patients starting with a three-minute warm up, forty-five minutes of aerobic training, resistance training, or combined exercise training, followed by a ten-minute cool down. The cool down was standardized, as every patient stopped their program and followed along to the same video presented on a screen. The cool down consisted of static stretches for each major muscle group and was held for five to ten seconds. Throughout the three months, patients followed their personalized routine under the guidance of the CR staff.

Exercise intensities for each patient were individualized based on the patient’s ability. Intensities were determined using the Borg Rating of Perceived Exertion Scale (RPE) [25], in which the patient chose a number on the RPE scale that they felt best reflected how strenuous they felt the exercise was and the level of exertion in which they were working. The Borg RPE scale begins at number six, which reflects the level of exertion when sitting or standing. The numbers six to eleven reflect a light amount of effort, twelve to fifteen reflect a moderate amount of exertion, and sixteen to twenty reflect working at maximal capacity. The goal was for patients to feel as though they were working in the twelve to fifteen range. For all aerobic and anerobic exercise, patients were encouraged to work at an exertion they felt reflected within the moderate intensity range. In addition to the RPE scale, heart rate was also monitored as a tool to gauge the patient’s effort. Resting heart rate was obtained for each patient prior to the start of each exercise session. Most patients were on beta-blocker medication, which lowers heart rate. RPE was the preferred method to assess intensity; however, patients’ heart rates were monitored and maintained within twenty to thirty beats per minute over their resting heart rate. Any exercise that was performed outside of CR was not accounted for the current study.

### 2.4. Aerobic Training

Patients engaged in either one or more of the following aerobic machines: arm ergometer, treadmill, stationary bike, recumbent bike, NuStep Recumbent Cross Trainer T4r [26], seated elliptical, or standing elliptical. For all machines, patients were required to stay between 50 and 60 revolutions per minute (RPM). If their exercise program included interval training, patients were required to stay between 80 and 100 RPM during the high-intensity intervals. Time spent on all machines varied for each patient, except for the arm ergometer, for which no patient exceeded five minutes. For the first few weeks of the program, each patient started with low-intensity training, but as the program continued, all patients used RPE and heart rate as a guide on how to increase their level of exertion. 

### 2.5. Resistance Training

Depending on physical ability, resistance training was incorporated into each patient’s training. Almost all patients worked with one or more of the following: dumbbells, weighted boxes, and resistance bands. All resistance training prescribed targeted the upper body musculature. Training routines consisted of eight to fifteen repetitions and one to two sets per exercise. For dumbbells, the weight was between two and fifteen pounds and those who used dumbbells performed dumbbell chest presses, dumbbell rows, or both. Patients that used resistance bands followed a routine that included chest presses, bicep curls, shoulder extensions, and external rotations. The bands were attached to the wall via metal hoops and carabiners and there were seven hooks such that patients could adjust the bands to their own height or level or resistance desired. There were five total bands, yellow being the lightest and ranging from 2 to 17 pounds depending on the height of the band. Next in resistance was green (ranging from 3 to 30 pounds), red (ranging from 3 to 40 pounds), blue (ranging from 3 to 56 pounds), and purple (ranging from 3 to 59 pounds). For weighted boxes, forty to fifty pounds were put into a wooden box which patients carried to their different stations throughout the hour. Only patients that had a lifting requirement for returning to work were assigned this. Patients progressed at varying times throughout their program, using RPE and heart rate as a guide. CABG patients were required to follow standard sternal precautions with resistance training due to having a sternotomy.

### 2.6. Balance Exercises

Because the sample population was mainly geriatric, some patients were prescribed balance exercises. These included sit-to-stands, seated or standing abduction, adduction, single-leg balance, heel lifts, tightrope walking, flamingo stands, banded toe-taps, and chair leg raises. Accordingly, patients were prescribed between two and five balance exercises for two to three sets, and eight to fifteen repetitions.

### 2.7. Sternal Precautions

CABG patients were required to follow standardized sternal precautions during their training program due to undergoing a sternotomy procedure. Precautions are set to decrease the risk of dissonance of the sternum [27]. Precautions include the following directions: do not push or pull through the arms, avoid movement that only uses one side of the body, do not elevate the arms over 90 degrees, do not lift objects heavier than 5 pounds, use a self-hugging position when coughing, avoid the use of arms when transferring from sitting to standing, and do not place the arms behind the back [28]. After the first month or six to eight weeks, patients were allowed to progress in the amount of weight being lifted if there were no complications with the sternum and no there was no presence of angina or dyspnea. 

### 2.8. Nutrition

During their intake, patients were encouraged to follow the Dietary Approaches to Stop Hypertension (DASH) diet [29]. With the DASH diet, patients were encouraged to have a low-sodium diet with increased amounts of fruit, vegetables, and whole grains [29]. The diet also included limiting dairy, saturated fats, and sugar [29].

### 2.9. Primary Outcome

The primary outcome was to evaluate the influence of the number CR sessions on health markers per population and primary diagnosis. Because all data were included from the discharge lab work, the differences in variables were compared by diabetes diagnosis, primary cardiac diagnosis (CABG or PCI), and number of sessions attended (twelve to twenty-four or twenty-five to thirty-six). Outcome health markers observed from intake to discharge were total cholesterol, HDL, LDL, triglycerides, systolic blood pressure, diastolic blood pressure, PHQ-9, DASI, and body mass. For diabetic patients only, fasting blood glucose and A1C were assessed.

### 2.10. Statistical Analysis

Outcome data were obtained from a hospital’s medical center outpatient data registry stored with the American Association of Cardiovascular and Pulmonary Rehabilitation. Data were analyzed using a mixed factorial four-way 2 × 2 × 2 × 2 ANOVA (population [diabetes vs. no diabetes] × primary cardiac diagnosis [PCI vs. CABG] × number of sessions attended [twelve to twenty-four vs. twenty-five to thirty-six] × time [pre vs. post]. Additionally, correlations between the number of rehabilitation sessions attended and the pre- to post-test change for each value were analyzed. Data are reported as mean ± SE. Once data were collected, all information was placed into JASP Software (version 0.17.1, JASP Team, Amsterdam, The Netherlands) for statistical analysis. Results were considered significant at *p* ≤ 0.05 and post hoc tests with Bonferroni correction were used when appropriate.

## 3. Results

### 3.1. Participant Characteristics

Table 1 presents the number of participants in each category per cardiac diagnoses, number of sessions attended, and diabetes category.

### 3.2. Total Cholesterol

**Figure 1:**
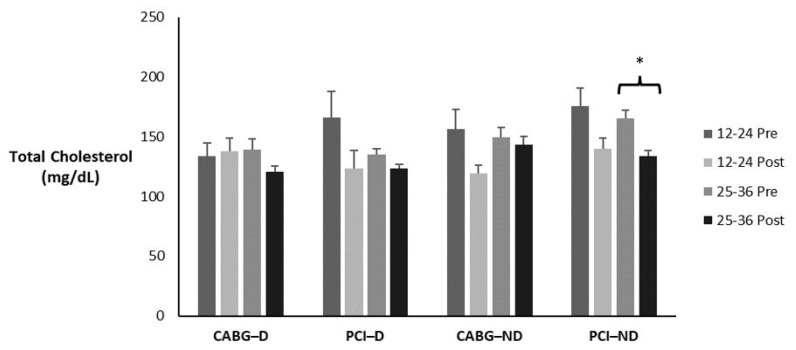
Means ± SE for total cholesterol (mg/dL). 12–24 = 12–24 sessions completed; 25–36 = 25–36 sessions completed; CABG = coronary artery bypass grafting; D = diabetic; mg/dL = milligrams per deciliter; ND = nondiabetic; PCI = percutaneous coronary intervention; Post = discharge values; Pre = intake values; * Denotes significant changes pre- to post-testing.

Table 2 and Figure 1 display the means ± SE and effect sizes for total cholesterol from pre- to post-test. Table 3 displays how many participants, regardless of condition, fell in each classification pre- to post-testing and the percent change. There was a significant four-way interaction for time × session group × diabetes category × admission event (*p* = 0.033). Post hoc testing revealed that the 25–36 PCI group with no diabetes had a significant decrease in total cholesterol (*p* < 0.001).

### 3.3. Triglycerides

**Figure 2:**
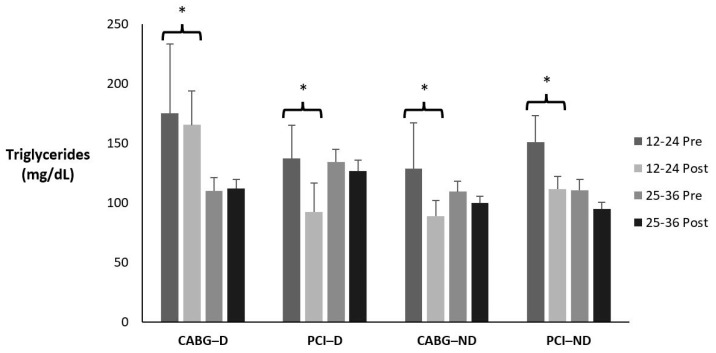
Means ± SE for triglycerides (mg/dL). 12–24 = 12–24 sessions completed; 25–36 = 25–36 sessions completed; CABG = coronary artery bypass grafting; D = diabetic; mg/dL = milligrams per deciliter; ND = nondiabetic; PCI = percutaneous coronary intervention; Post = discharge values; Pre = intake values; * Denotes significant changes pre- to post-testing.

Table 4 and Figure 2 display the means ± SE and effect sizes for triglycerides from pre- to post-test. Table 5 displays how many participants, regardless of condition, fell in each classification pre- to post-testing and the percent change. There was a two-way interaction for time × session group (*p* = 0.034). Post hoc testing revealed that the 12–24 session groups had a significant decrease in triglycerides (*p* = 0.015).

### 3.4. High-Density Lipoprotein

**Figure 3:**
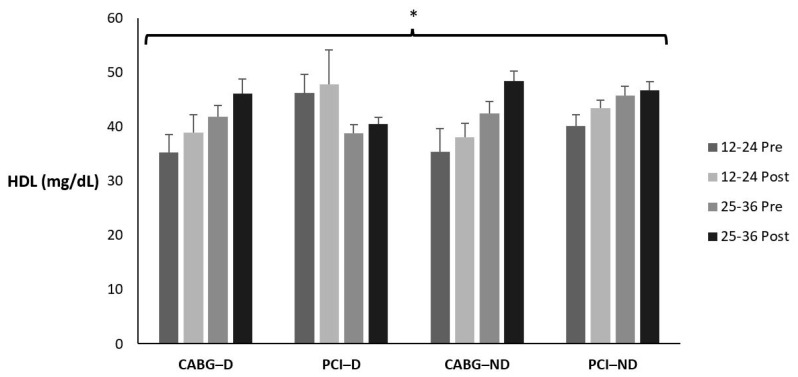
Means ± SE for high-density lipoprotein (mg/dL). 12–24 = 12–24 sessions completed; 25–36 = 25–36 sessions completed; CABG = coronary artery bypass grafting; D = diabetic; mg/dL = milligrams per deciliter; ND = nondiabetic; PCI = percutaneous coronary intervention; Post = discharge values; Pre = intake values; * Denotes significant changes pre- to post-testing.

Table 6 and Figure 3 display the means ± SE and effect sizes for HDL from pre- to post-test. Table 7 displays how many participants, regardless of condition, fell in each classification pre- to post-testing and the percent change. There was no four-way interaction for time × session group × diabetes category × admission event (*p* = 0.480). There were no three-way interactions for time × session group × diabetes category (*p* = 0.962), time × session group × admission event (*p* = 0.419), or time × diabetes category × admission event (*p* = 0.992). There were no two-way interactions for time × session group (*p* = 0.846), time × diabetes category (*p* = 0.822), or time × admission event (*p* = 0.216). However, there was a significant main effect for time (*p* = 0.001). HDL increased from pre- to post-test for all groups (*p* < 0.001). There were no other main effects for session group, diabetes category, or admission event (*p* = 0.160; *p* = 0.779; *p* = 0.213; respectively).

### 3.5. Low-Density Lipoprotein

**Figure 4:**
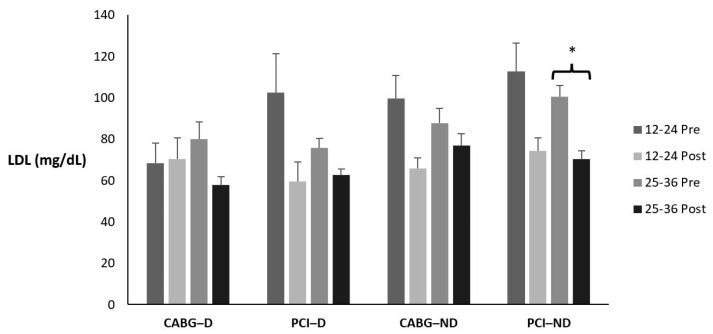
Means ± SE for low-density lipoprotein (mg/dL). 12–24 = 12–24 sessions completed; 25–36 = 25–36 sessions completed; CABG = coronary artery bypass grafting; D = diabetic; mg/dL = milligrams per deciliter; ND = nondiabetic; PCI = percutaneous coronary intervention; Post = discharge values; Pre = intake values; * Denotes significant changes pre- to post-testing.

Table 8 and Figure 4 display the means ± SE and effect sizes for LDL from pre- to post-test. Table 9 displays how many participants, regardless of condition, fell in each classification pre- to post-testing and the percent change. There was a four-way interaction for time × session group × diabetes category × admission event (*p* = 0.038). Post hoc testing revealed that the 25–36 nondiabetic PCI group had a significant decrease in LDL (*p* < 0.001).

### 3.6. Systolic Blood Pressure

**Figure 5:**
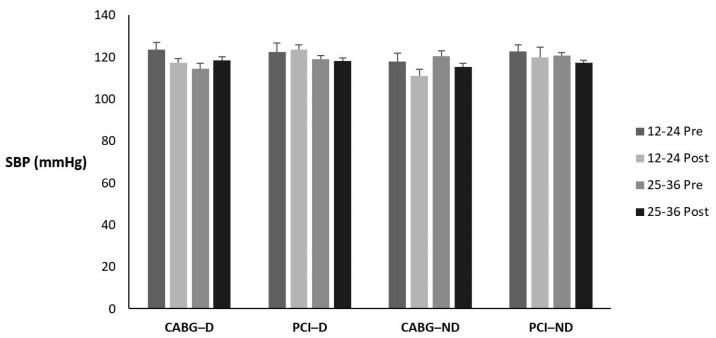
Means ± SE for systolic blood pressure (mmHg). 12–24 = 12–24 sessions completed; 25–36 = 25–36 sessions completed; CABG = coronary artery bypass grafting; D = diabetic; mmHg = milliliters of mercury; ND = nondiabetic; PCI = percutaneous coronary intervention; Post = discharge values; Pre = intake values.

Table 10 and Figure 5 display the means ± SE and effect sizes for systolic blood pressure from pre- to post-test. Table 11 displays how many participants, regardless of condition, fell in each classification pre- to post-testing and the percent change. There was no four-way interaction for time × session group × diabetes category × admission event (*p* = 0.372). There were no three-way interactions for time × session group × diabetes category (*p* = 0.493), time × session group × admission event (*p* = 0.171), or time × diabetes category × admission event (*p* = 0.802). There were no two-way interactions for time × session group (*p* = 0.384), time × diabetes category (*p* = 0.129), or time × admission event (*p* = 0.441). There were no main effects for time, session group, diabetes category, or admission event (*p* = 0.063; *p* = 0.336; *p* = 0.450; *p* = 0.106; respectively).

### 3.7. Diastolic Blood Pressure

**Figure 6:**
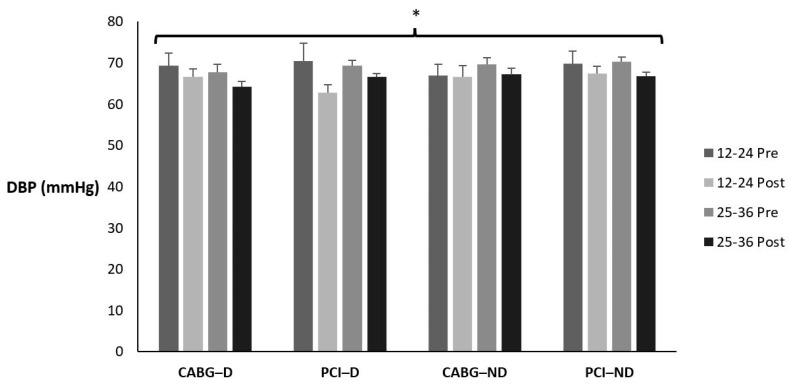
Means ± SE for diastolic blood pressure (mmHg). 12–24 = 12–24 sessions completed; 25–36 = 25–36 sessions completed; CABG = coronary artery bypass grafting; D = diabetic; mmHg = milliliters of mercury; ND = nondiabetic; PCI = percutaneous coronary intervention; Post = discharge values; Pre = intake values. * Denotes significant changes pre- to post-testing.

Table 12 and Figure 6 display the means ± SE and effect sizes for diastolic blood pressure from pre- to post-test. Table 13 displays how many participants, regardless of condition, fell in each classification pre- to post-testing and the percent change. There was no four-way interaction for time × session group × diabetes category × admission event (*p* = 0.615). There were no three-way interactions for time × session group × diabetes category (*p* = 0.419), time × session group × admission event (*p* = 0.434), or time × diabetes category × admission event (*p* = 0.912). There were no two-way interactions for time × session group (*p* = 0.950), time × diabetes category (*p* = 0.381), or time × admission event (*p* = 0.408). However, there was a significant main effect for time (*p* = 0.004). Diastolic blood pressure decreased from pre- to post-test for all groups (*p* = 0.004). There were no other main effects for session group, diabetes category, or admission event (*p* = 0.856; *p* = 0.472; *p* = 0.624; respectively).

### 3.8. Body Mass

**Figure 7:**
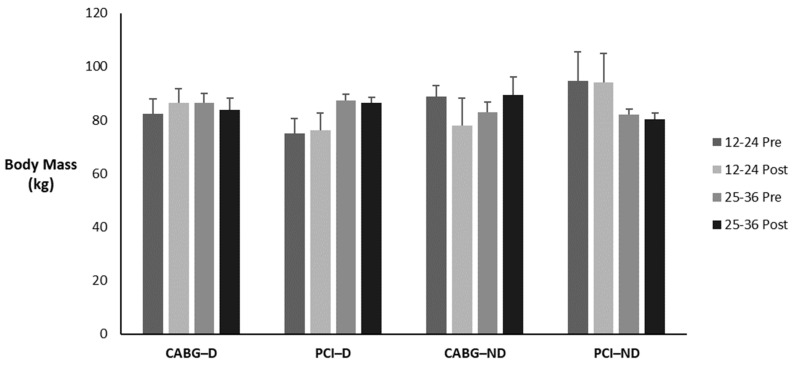
Means ± SE for body mass (kg). 12–24 = 12–24 sessions completed; 25–36 = 25–36 sessions completed; CABG = coronary artery bypass grafting; D = diabetic; kg = kilogram; ND = nondiabetic; PCI = percutaneous coronary intervention; Post = discharge values; Pre = intake values.

Table 14 and Figure 7 display the means ± SE and effect sizes for body mass from pre- to post-test. There was no four-way interaction for time × session group × diabetes category × admission event (*p* = 0.132). There were no three-way interactions for time × session group × diabetes category (*p* = 0.094), time × session group × admission event (*p* = 0.207), or time × diabetes category × admission event (*p* = 0.975). There were no two-way interactions for time × session group (*p* = 0.448), time × diabetes category (*p* = 0.690), or time × admission event (*p* = 0.784). There were no main effects for time, session group, diabetes category, or admission event (*p* = 0.534; *p* = 0.993; *p* = 0.481; *p* = 0.885; respectively).

### 3.9. Duke Activity Score Index

**Figure 8:**
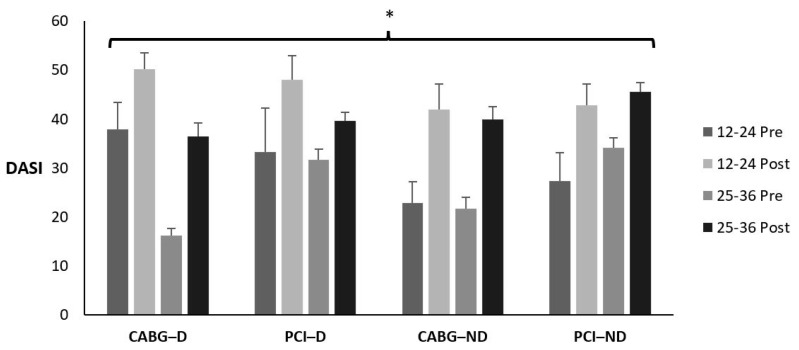
Means ± SE for the Duke Activity Score Index. 12–24 = 12–24 sessions completed; 25–36 = 25–36 sessions completed; CABG = coronary artery bypass grafting; D = diabetic; ND = nondiabetic; PCI = percutaneous coronary intervention; Post = discharge values; Pre = intake values. * Denotes significant changes pre- to post-testing.

Table 15 and Figure 8 display the means ± SE and effect sizes for the Duke Activity Score Index from pre- to post-test. There was no four-way interaction for time × session group × diabetes category × admission event (*p* = 0.309). There were no three-way interactions for time × session group × diabetes category (*p* = 0.592), time × session group × admission event (*p* = 0.117), or time × diabetes category × admission event (*p* = 0.989). There were no two-way interactions for time × session group (*p* = 0.735), time × diabetes category (*p* = 0.415), or time × admission event (*p* = 0.079). However, there was a significant main effect for time (*p* < 0.001). The Duke Activity Score Index increased from pre- to post-test for all groups (*p* < 0.001). There were no other main effects for session group, diabetes category, or admission event (*p* = 0.067; *p* = 0.420; *p* = 0.098; respectively).

### 3.10. Patient Health Questionnaire-9

**Figure 9:**
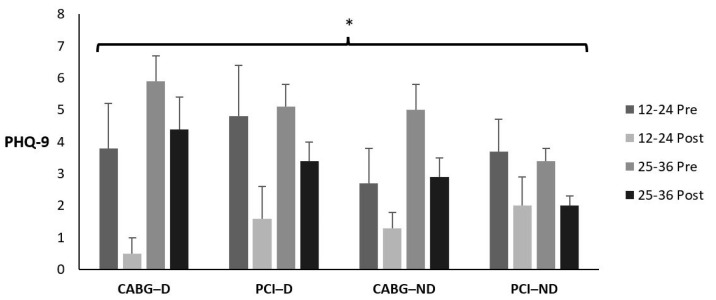
Means ± SE for the Patient Health Questionnaire-9. 12–24 = 12–24 sessions completed; 25–36 = 25–36 sessions completed; CABG = coronary artery bypass grafting; D = diabetic; ND = nondiabetic; PCI = percutaneous coronary intervention; Post = discharge values; Pre = intake values. * Denotes significant changes pre- to post-testing.

Table 16 and Figure 9 display the means ± *SE* and effect sizes for the PHQ-9 from pre- to post-test. There was no four-way interaction for time × session group × diabetes category × admission event (*p* = 0.662). There were no three-way interactions for time × session group × diabetes category (*p* = 0.211), time × session group × admission event (*p* = 0.821), or time × diabetes category × admission event (*p* = 0.877). There were no two-way interactions for time × session group (*p* = 0.302), time × diabetes category (*p* = 0.263), or time × admission event (*p* = 0.897). However, there was a significant main effect for time (*p* < 0.001). PHQ-9 decreased from pre- to post-test for all groups (*p* < 0.001). There were no other main effects for session group, diabetes category, or admission event (*p* = 0.049; *p* = 0.272; *p* = 0.936; respectively).

### 3.11. Fasting Blood Glucose

**Figure 10:**
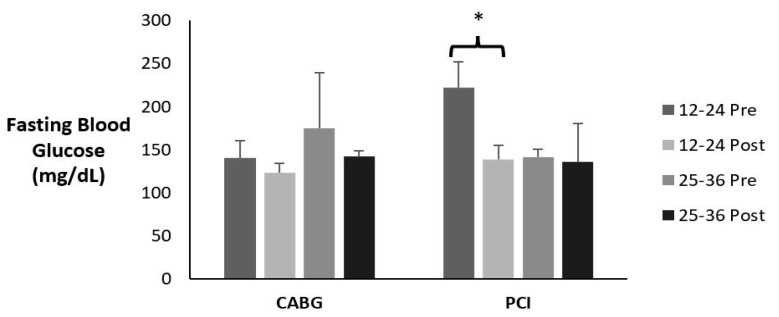
Means ± SE for fasting blood glucose (mg/dL). 12–24 = 12–24 sessions completed; 25–36 = 25–36 sessions completed; CABG = coronary artery bypass grafting; mg/dL = milligrams per deciliter; PCI = percutaneous coronary intervention; Post = discharge values; Pre = intake values. * Denotes significant changes pre- to post-testing.

Table 17 and Figure 10 display the means ± SE and effect sizes for fasting blood glucose from pre- to post-test, for the diabetic patients. There was a three-way interaction for time × session group × admission event (*p* = 0.013). Post hoc testing revealed that the 12–24 PCI group with diabetes had a significant decrease in fasting blood glucose (*p* = 0.037).

### 3.12. A1c

**Figure 11:**
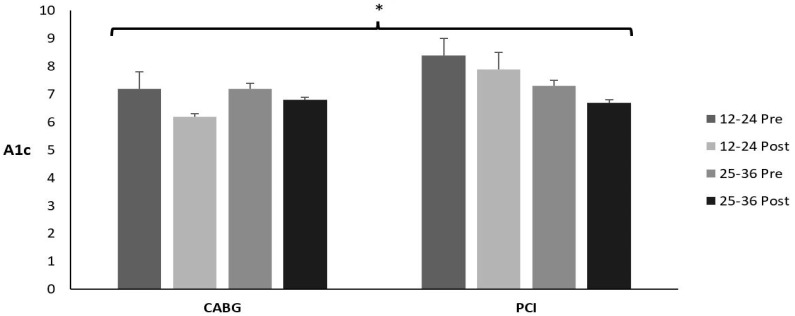
Means ± SE for A1c. 12–24 = 12–24 sessions completed; 25–36 = 25–36 sessions completed; CABG = coronary artery bypass grafting; PCI = percutaneous coronary intervention; Post = discharge values; Pre = intake values. * Denotes significant changes pre- to post-testing.

Table 18 and Figure 11 display the means ± SE and effect sizes for A1c from pre- to post-test, for the diabetic patients only. There was no three-way interaction for time × session group × admission event (*p* = 0.480). There were no two-way interactions for time × session group (*p* = 0.479) or time × admission event (*p* = 0.871). However, there was a significant main effect for time (*p* = 0.003). A1c decreased from pre- to post-test for both groups (*p* = 0.003). There were no other main effects for session group or admission events (*p* = 0.161; *p* = 0.021; respectively).

### 3.13. Overall Change across Each Variable

**Figure 12:**
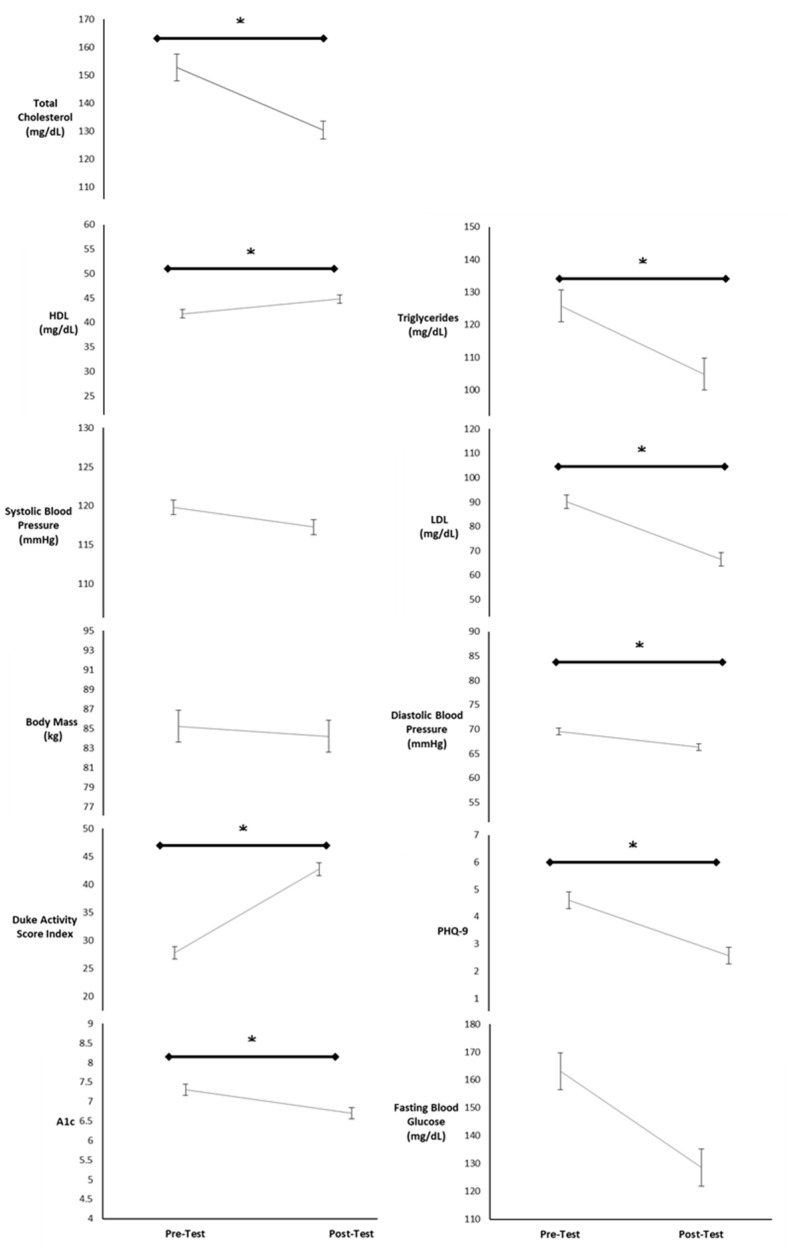
Means ± SE from pre- to post-test collapsed across cardiovascular, diabetes diagnoses, and session groups. mg/dL = milligrams per deciliter; mmHg = milliliters of mercury; kg = kilogram; pre = intake values; post = discharge values. * Denotes significant changes pre- to post-testing.

Figure 12 displays the overall change from pre-test to post-test for total cholesterol, triglycerides, HDL, LDL, systolic blood pressure, diastolic blood pressure, body mass, PHQ-9, DASI, fasting blood glucose, and A1c collapsed across cardiovascular, diabetes diagnoses, and session groups.

### 3.14. Percent Change Collapsed across All Variables

Table 19 displays percent change for means ± SE from pre- to post-test collapsed across cardiovascular and diabetes diagnoses, as well as session groups, for total cholesterol, triglycerides, HDL, LDL, systolic blood pressure, diastolic blood pressure, body mass, PHQ-9, DASI, fasting blood glucose, and A1c.

### 3.15. Pearson’s Correlations

Table 20 and Table 21 display the correlations between the number of rehabilitation sessions attended and the pre- to post-test change for each value. Table 14 is for all patients and Table 15 is for the diabetic patients.

## 4. Discussion

The primary results of the study indicate there were significant findings pre- to post-testing for time for HDL, diastolic blood pressure, the DASI, PHQ-9, and A1c. Systolic blood pressure, body mass, and fasting blood glucose results did not indicate a significant difference. Time, session group, diabetes category, and admission event influenced total cholesterol and LDL for the 25–36 PCI nondiabetic group. Time and session group influenced triglycerides for all 12–24 session groups. HDL significantly increased for all groups. Systolic blood pressure did not change and diastolic blood pressure significantly decreased. There was no change in body mass for all groups. The DASI scores significantly increased while the PHQ-9 scores significantly decreased. Time, session group, and admission event had an influence on the PCI with diabetes group, significantly decreasing fasting blood glucose. A1c also significantly decreased for pre- to post-testing.

These changes are consistent with previous research [14,30]. Mann et al. [14] reviewed evidence from thirteen studies which observed the effects of combined aerobic and anaerobic training on total cholesterol and the lipid panel. Results comprehensively revealed improvements in total cholesterol, triglycerides, HDL, and LDL, which are consistent with the current study [14]. Findings may be attributed to the type of training given to individuals and the consistent dose–response relationship found with physical activity and lipid improvement [30]. Aadahl et al. [30] conducted a cross-sectional study which observed a dose–response relationship, through questionnaires and biological variables. They reported the level of one’s activity was directly correlated with their lab results, consisting of total cholesterol, triglycerides, HDL, LDL, BMI, and waist-to-hip ratio [30]. Justification for the current study can be observed by the increase in DASI scores among all patient groups. Patients reported higher levels of physical activity and functionality following completion of cardiac rehabilitation compared to the initial intake.

In a meta-analysis by Chen et al. [31], similar cholesterol improvements to the current study were reported. Eighteen studies, which were comprised of exercise intervention, including but not limited to cycling, walking, running, and arm ergometer training, were used [31]. The meta-analysis revealed similar findings with HDL and LDL, but in contrast to the current study, they found a significant improvement in systolic blood pressure [31]. In addition to these findings, Kargarfard et al. [32] observed blood pressure in cardiac patients for a duration of two months. Subjects exercised for similar durations as those in the present study with a warm up, thirty minutes of exercise, and a cool down. Aerobic training consisted of walking and running on a treadmill. Exercise rehabilitation revealed improvements in both systolic and diastolic blood pressures [32]. These findings were congruent with the diastolic blood pressure results of the current study. The differences in systolic blood pressure findings between Chen et al. [31], Kargarfard et al. [32], and the current study could be attributed to the differences in cardiac diagnoses, cardiac medications each study sample was taking, or the potential for error when blood pressure is measured [33]. With these differences, the Cleveland Clinic [34] reported similar results for systolic blood pressure as those of the current study. Similarly to the Cleveland Clinic [34], the present study did not see improvements in systolic blood pressure.

For both questionnaires, significant results were found. The DASI had significant improvements for all groups pre- to post-test. Increases in the DASI score reflect the patients’ increased perception of their capability to do daily tasks [35]. The results from the current study are comparable to those of a study by Bhattal et al. [35], which reported significant improvements in the DASI with home-based cardiac rehabilitation. Patients followed similar exercise prescriptions for both aerobic and anerobic exercise, similarly to the current study. For the PHQ-9, the mean score for all groups significantly decreased. These results were similar to those of the Cardiac Rehabilitation Center at the Cleveland Clinic [34]. They found through rehabilitation implementation that there was a significant decrease in PHQ-9 scores [34]. Decreases in the PHQ-9 score reflect a decrease in symptoms of depression. There is limited research on the usage of these questionnaires in cardiac rehabilitation settings, which is a benefit of the current study to potentially add to the existing research and literature.

Body mass decreased throughout cardiac rehabilitation, but this was not significant. This is similar to a Cleveland Clinic study [34] demonstrating a non-significant increase in body mass throughout the duration of their program. A study conducted by Barrett et al. [36] reported different results to those of the current study. Within their population, they split the patients into two groups: those who wished to decrease body mass and those who did not. Those whose goal was to achieve weight loss during CR had a significant decrease in body mass [36]. Those who did not wish to lose body mass, did not have a significant change [36]. Therefore, demonstrating the clinical setting may not be the main component in achieving a reduction in body mass. There are a few possible reasons for this discrepancy. Age may have played a role, as older patients within this study were uninterested in weight loss or dietary change. Frail older patients may have prioritized weight gain as a goal. The sample may have had more patients who exhibited lack of motivation or high levels of sedentary time. A large majority, 43%, of the participants was affected by the COVID-19 pandemic, in which sedentary time significantly increased [37] and changed the day-to-day lifestyle of many patients. These patterns in lack of motivation and at-home sedentary time could have continued throughout attending the CR program. In addition, poor nutrition and lack of dietary recommendations could have occurred. Additionally, muscle gain from anaerobic training could have affected the lack of body mass change.

A1c had significant improvements for the diabetic groups throughout CR. Similar results were reported in a meta-analysis conducted by Wu et al. [38]. Vast statistical reductions in A1c were found through consistent exercise trials, favoring the intervention of exercise [38]. Individuals that participated in an intervention that was three or more days per week saw an overall higher reduction than those who participated in the less frequent exercise intervention [38]. These results were also in accordance with those of Wang et al. [39], who demonstrated that with exercise and health education, patients had significant reductions in A1c. For fasting blood glucose, no significant changes were found. These results are similar to those in a study conducted by Bennasar-Venny et al. [40], which found no significant change in fasting blood glucose with aerobic training. Resistance and interval training were shown to significantly lower fasting blood glucose, but the researchers found that the strength of the majority of the evidence was low [40]. Norton et al. [41] reported that with consistent exercise training, the body was able to upregulate glucose control, which contributed to proficient numbers [41]. Patients had significantly lower fasting blood glucose at the end of the study [41]. These changes could indicate the body’s ability to upregulate glucose with consistent, combined training of both aerobic and anaerobic exercise. Therefore, the lack of change for fasting blood glucose of the current study could be attributed to the way the statistics were analyzed. Bennasar-Venny et al. [40] and Norton et al. [41] analyzed the upregulation of glucose, observing how those with low glucose readings increased and those with high glucose readings decreased. Therefore, the results showed that through exercise, the body was able to better manage glucose.

One limitation of this study is that the number of patients for each group was different. The smaller groups may have suffered due to a lack of power. Due to having one larger group, small differences among the data may have not been observed. A second limitation of this data set is that age and comorbidities were not used in the filtering of the data. This could have altered the data, as a large number of patients have comorbidities or barriers due to age or physical limitations. Future research could account for these variables and their influence on health markers and performance outcomes. A third limitation of this study was not being able to filter the data by the patient’s exercise routine. Because each patient had an individualized routine, some patients may have been working at higher workloads than others. Future research could include the influence of following a specific exercise regimen on health makers and performance outcomes. Additionally, future research could include additional cardiac diagnoses, expanding the influence of CR to various cardiac populations. Lastly, an option would be to equate group sizes, removing influences from unequal group sample sizes. Although patients were prescribed 36 sessions, many patients did not complete all 36 sessions for various reasons. These reasons included, but were not limited to, lack of transportation, lack of desire to exercise, personal finances, motivation, or comorbidities, or patients were previously conditioned and healthy before their event. Future recommendations for CR programs could include using a body composition assessment in order to evaluate fat mass and fat-free mass changes, or lack thereof, throughout the program. This would also help to comprehensively evaluate any body mass change from pre- to post-testing. Programs may also want to monitor each patient’s diet, in order to specifically evaluate the role of patient nutrition with their changes in body composition, lipid panel, and vitals throughout the program.

In summary, cardiac rehab was found to be safe and effective for cardiac patients. Significant interactions were found for total cholesterol, triglycerides, HDL, LDL, diastolic blood pressure, the DASI, PHQ-9, and A1c, therefore revealing that CR had a positive influence on health markers and performance outcomes, regardless of which subgroup they were in. It can be concluded that the number of CR sessions attended was not a determinant in patient improvement. Furthermore, the significant changes in both questionnaires satisfied the alternative secondary hypothesis, which stated that all groups would show improvement regardless of the number of CR sessions attended.

## Figures and Tables

**Table 1:** Participant characteristics.

Condition	Diabetes	No Diabetes	Total
12–24 Sessions	*N* = 11	*N* = 17	*N* = 28
CABG	6	7	13
PCI	5	10	15
25–36 Sessions	*N* = 77	*N* = 92	*N* = 169
CABG	24	32	56
PCI	53	60	113

Abbreviation: 12–24 Sessions = 12–24 sessions completed; 25–36 Sessions = 25–36 sessions completed; CABG = coronary artery bypass grafting; diabetes = type 1 and type 2 diabetes; PCI = percutaneous coronary intervention.

**Table 2:** Means ± SE for total cholesterol (mg/dL).

Condition	Pre	Post	Cohen’s d	% Change
12–24 Sessions				
CABG with Diabetes	134.0 ± 10.9	138.0 ± 11.0	0.1	2.9%
PCI with Diabetes	166.0 ± 22.3	123.8 ± 14.5	1.1	−25.4%
CABG with No Diabetes	156.7 ± 16.1	119.7 ± 6.7	1.0	−23.6%
PCI with No Diabetes	175.8 ± 14.7	140.1 ± 8.9	0.9	−20.3%
25–36 Sessions				
CABG with Diabetes	139.0 ± 9.0	120.8 ± 4.7	0.5	−13.0%
PCI with Diabetes	135.1 ± 5.2	123.3 ± 3.4	0.3	−8.7%
CABG with No Diabetes	149.3 ± 8.5	143.4 ± 6.7	0.2	−3.9%
PCI with No Diabetes	165.7 ± 6.4	134.0 ± 4.7 ^a^	0.8	−19.1%

^a^ Denotes significant changes pre- to post-testing. Abbreviation: 12–24 Sessions = 12–24 sessions completed; 25–36 Session = 25–36 sessions completed; CABG = coronary artery bypass grafting; diabetes = type 1 and type 2 diabetes; mg/dL = milligrams per deciliter; PCI = percutaneous coronary intervention.

**Table 3:** Classifications and percent change for total cholesterol.

Classification	Pre	Post	Percent Change
Desirable	*N* = 165	*N* = 192	16%
Borderline High	*N* = 25	*N* = 3	−88%
High	*N* = 7	*N* = 2	−71%

Abbreviation: Desirable = <200 mg/dL; Borderline High = 200–239 mg/dL; High = >240 mg/dL [24].

**Table 4:** Means ± SE for triglycerides (mg/dL).

Condition	Pre	Post	Cohen’s d	% Change
12–24 Sessions				
CABG with Diabetes	175.3 ± 57.9	165.3 ± 28.5 ^a^	0.2	−5.7%
PCI with Diabetes	137.0 ± 27.9	92.2 ± 24.1 ^a^	0.7	−32.7%
CABG with No Diabetes	128.8 ± 38.4	88.8 ± 13.2 ^a^	0.7	−31.0%
PCI with No Diabetes	150.7 ± 22.7	111.3 ± 10.9 ^a^	0.6	−26.1%
25–36 Sessions				
CABG with Diabetes	110.1 ± 11.2	110.0 ± 7.6	0.0	0.0%
PCI with Diabetes	134.4 ± 10.2	126.5 ± 9.2	0.1	−5.8%
CABG with No Diabetes	109.2 ± 8.9	99.7 ± 5.6	0.2	−8.7%
PCI with No Diabetes	110.4 ± 9.0	94.7 ± 5.6	0.3	−14.2%

^a^ Denotes significant changes pre- to post-testing. Abbreviation: 12–24 Sessions = 12–24 sessions completed; 25–36 Session = 25–36 sessions completed; CABG = coronary artery bypass grafting; diabetes = type 1 and type 2 diabetes; mg/dL = milligrams per deciliter; PCI = percutaneous coronary intervention.

**Table 5:** Classifications and percent change for triglycerides.

Classification	Pre	Post	Percent Change
Normal	*N* = 146	*N* = 166	14%
Borderline High	*N* = 27	*N* = 17	−37%
High	*N* = 24	*N* = 14	−42%
Very High	*N* = 0	*N* = 0	0%

Abbreviation: Normal = <150 mg/dL; Borderline High = 150–199 mg/dL; High = 200–499 mg/dL; Very High = >500 mg/dL [21].

**Table 6:** Means ± SE for high-density lipoprotein (mg/dL).

Condition	Pre	Post	Cohen’s d	% Change
12–24 Sessions				
CABG with Diabetes	35.3 ± 3.3	39.0 ± 3.3 ^a^	0.3	10.4%
PCI with Diabetes	46.2 ± 3.5	47.8 ± 6.4 ^a^	0.1	3.4%
CABG with No Diabetes	35.4 ± 4.3	38.1 ± 2.5 ^a^	0.2	7.6%
PCI with No Diabetes	40.2 ± 2.1	43.4 ± 1.5 ^a^	0.3	7.9%
25–36 Sessions				
CABG with Diabetes	41.9 ± 2.1	46.1 ± 2.7 ^a^	0.4	10%
PCI with Diabetes	38.8 ± 1.6	40.5 ± 1.3 ^a^	0.1	4.3%
CABG with No Diabetes	42.5 ± 2.2	48.5 ± 1.8 ^a^	0.5	14.1%
PCI with No Diabetes	45.8 ± 1.7	46.7 ± 1.6 ^a^	0.1	1.9%

^a^ Denotes significant changes pre- to post-testing. Abbreviation: 12–24 Sessions = 12–24 sessions completed; 25–36 Session = 25–36 sessions completed; CABG = coronary artery bypass grafting; diabetes = type 1 and type 2 diabetes; mg/dL = milligrams per deciliter; PCI = percutaneous coronary intervention.

**Table 7:** Classifications and percent change for high-density lipoprotein.

Classification	Pre	Post	Percent Change
Low	*N* = 87	*N* = 70	−20%
Desirable	*N* = 110	*N* = 127	15%

Abbreviation: Low = <40 mg/dL; Desirable = ≥40 mg/dL [21].

**Table 8:** Means ± SE for low-density lipoprotein (mg/dL).

Condition	Pre	Post	Cohen’s d	% Change
12–24 Sessions				
CABG with Diabetes	68.3 ± 9.8	70.3 ± 10.4	0.1	2.9%
PCI with Diabetes	102.6 ± 18.7	59.6 ± 9.5	1.3	−41.9%
CABG with No Diabetes	99.6 ± 11.1	65.8 ± 5.2	1.0	−33.9%
PCI with No Diabetes	112.9 ± 13.5	74.4 ± 6.3	1.2	−34.1%
25–36 Sessions				
CABG with Diabetes	80.1 ± 8.3	57.8 ± 4.1	0.7	−27.8%
PCI with Diabetes	75.9 ± 4.4	62.6 ± 2.9	0.4	−17.5%
CABG with No Diabetes	87.8 ± 7.0	76.8 ± 5.7	0.3	−12.5%
PCI with No Diabetes	100.6 ± 5.3	70.5 ± 3.9 ^a^	0.9	−29.9%

^a^ Denotes significant changes pre- to post-testing. Abbreviation: 12–24 Sessions = 12–24 sessions completed; 25–36 Session = 25–36 sessions completed; CABG = coronary artery bypass grafting; diabetes = type 1 and type 2 diabetes; mg/dL = milligrams per deciliter; PCI = percutaneous coronary intervention.

**Table 9:** Classifications and percent change for low-density lipoprotein.

Classification	Pre	Post	Percent Change
Desirable	*N* = 124	*N* = 175	41%
Above Desirable	*N* = 41	*N* = 16	−61%
Borderline High	*N = 25*	*N* = 5	−80%
High	*N* = 3	*N* = 1	−67%
Very High	*N* = 4	*N* = 0	−100%

Abbreviation: Desirable = <100 mg/dL; Above Desirable = 100–129 mg/dL; Borderline High = 130–159 mg/dL; High = 160–189 mg/dL; Very High = >190 mg/dL [21].

**Table 10:** Means ± SE for systolic blood pressure (mmHg).

Condition	Pre	Post	Cohen’s d	% Change
12–24 Sessions				
CABG with Diabetes	123.7 ± 3.3	117.3 ± 2.1	0.6	−5.1%
PCI with Diabetes	122.4 ± 4.4	123.6 ± 2.2	0.1	0.9%
CABG with No Diabetes	118.0 ± 3.8	111.1 ± 3.1	0.6	−5.8%
PCI with No Diabetes	122.8 ± 3.2	120.0 ± 4.7	0.3	−2.2%
25–36 Sessions				
CABG with Diabetes	114.5 ± 2.6	118.5 ± 1.6	0.4	3.4%
PCI with Diabetes	119.0 ± 1.7	118.3 ± 1.3	0.1	−0.5%
CABG with No Diabetes	120.4 ± 2.5	115.3 ± 1.8	0.5	−4.2%
PCI with No Diabetes	120.7 ± 1.6	117.2 ± 1.3	0.3	−2.9%

Abbreviations: 12–24 Sessions = 12–24 sessions completed; 25–36 Sessions = 25–36 sessions completed; CABG = coronary artery bypass grafting; diabetes = type 1 and type 2 diabetes; mmHg = milliliters of mercury; PCI = percutaneous coronary intervention.

**Table 11:** Classifications and percent change for systolic blood pressure.

Classification	Pre	Post	Percent Change
Normal	*N* = 114	*N* = 131	15%
Elevated	*N* = 36	*N* = 52	44%
Hypertension Stage 1	*N* = 37	*N* = 11	−70%
Hypertension Stage 2	*N* = 10	*N* = 3	−70%

Abbreviation: Normal = <120 mmHg; Elevated = 120–129 mmHg; Hypertension Stage 1 = 130–139 mmHg; Hypertension Stage 2 = >140 mmHg [21].

**Table 12:** Means ± SE for diastolic blood pressure (mmHg).

Condition	Pre	Post	Cohen’s d	% Change
12–24 Sessions				
CABG with Diabetes	69.3 ± 3.1	66.7 ± 1.8 ^a^	0.3	−3.7%
PCI with Diabetes	70.4 ± 4.4	62.8 ± 1.9 ^a^	0.9	−10.7%
CABG with No Diabetes	66.9 ± 2.8	66.6 ± 2.7 ^a^	0.0	0.4%
PCI with No Diabetes	69.9 ± 3.0	67.4 ± 1.8 ^a^	0.3	−3.5%
25–36 Sessions				
CABG with Diabetes	67.8 ± 1.8	64.2 ± 1.4 ^a^	0.4	−5.3%
PCI with Diabetes	69.4 ± 1.3	66.6 ± 0.9 ^a^	0.3	−4.0%
CABG with No Diabetes	69.7 ± 1.5	67.2 ± 1.5 ^a^	0.3	−3.5%
PCI with No Diabetes	70.3 ± 1.1	66.8 ± 1.0 ^a^	0.4	−4.9%

^a^ Denotes significant changes pre- to post-testing. Abbreviations: 12–24 Sessions = 12–24 sessions completed; 25–36 Sessions = 25–36 sessions completed; CABG = coronary artery bypass grafting; diabetes = type 1 and type 2 diabetes; mmHg = milliliters of mercury; PCI = percutaneous coronary intervention.

**Table 13:** Classifications and percent change for diastolic blood pressure.

Classification	Pre	Post	Percent Change
Normal/Elevated	*N* = 155	*N* = 180	16%
Hypertension Stage 1	*N* = 39	*N* = 17	−56%
Hypertension Stage 2	*N* = 3	*N* = 0	−100%

Abbreviation: Normal/ Elevated = <80 mmHg; Hypertension Stage 1 = 80–89 mmHg; Hypertension Stage 2 = >90 mmHg [21].

**Table 14:** Means ± SE for body mass (kg).

Condition	Pre	Post	Cohen’s d	% Change
12–24 Sessions				
CABG with Diabetes	82.3 ± 5.7	86.4 ± 5.2	0.1	4.9%
PCI with Diabetes	75.1 ± 5.6	76.3 ± 6.2	0.1	1.5%
CABG with No Diabetes	88.8 ± 4.0	77.9 ± 10.2	0.5	−12.2%
PCI with No Diabetes	94.8 ± 10.8	94.2 ± 10.6	0.0	−0.6%
25–36 Sessions				
CABG with Diabetes	86.4 ± 3.7	83.7 ± 4.4	0.1	−3.1%
PCI with Diabetes	87.3 ± 2.4	86.3 ± 2.3	0.1	−1.1%
CABG with No Diabetes	83.0 ± 3.6	89.5 ± 6.7	0.3	7.8%
PCI with No Diabetes	82.0 ± 2.1	80.3 ± 2.3	0.1	−2.0%

Abbreviations: 12–24 Sessions = 12–24 sessions completed; 25–36 Sessions = 25–36 sessions completed; CABG = coronary artery bypass grafting; diabetes = type 1 and type 2 diabetes; kg = kilogram; PCI = percutaneous coronary intervention.

**Table 15:** Means ± SE for the Duke Activity Score Index.

Condition	Pre	Post	Cohen’s d	% Change
12–24 Sessions				
CABG with Diabetes	37.9 ± 5.5	50.2 ± 3.3 ^a^	0.9	32.4%
PCI with Diabetes	33.3 ± 8.9	48.0 ± 4.9 ^a^	1.0	44.1%
CABG with No Diabetes	22.9 ± 4.3	41.9 ± 5.3 ^a^	1.3	82.9%
PCI with No Diabetes	27.3 ± 5.8	42.9 ± 4.3 ^a^	1.1	57.1%
25–36 Sessions				
CABG with Diabetes	16.2 ± 1.4	36.5 ± 2.7 ^a^	1.4	125%
PCI with Diabetes	31.7 ± 2.2	39.6 ± 1.8 ^a^	1.2	24.9%
CABG with No Diabetes	21.7 ± 2.3	39.9 ± 2.7 ^a^	1.3	83.8%
PCI with No Diabetes	34.1 ± 2.1	45.6 ± 1.8 ^a^	0.8	33.7%

^a^ Denotes significant changes pre- to post-testing. Abbreviations: 12–24 Sessions = 12–24 sessions completed; 25–36 Sessions = 25–36 sessions completed; CABG = coronary artery bypass grafting; diabetes = type 1 and type 2 diabetes; PCI = percutaneous coronary intervention.

**Table 16:** Means ± SE for the Patient Health Questionnaire-9.

Condition	Pre	Post	Cohen’s d	% Change
12–24 Sessions				
CABG with Diabetes	3.8 ± 1.4	0.5 ± 0.5 ^a^	0.9	−87%
PCI with Diabetes	4.8 ± 1.6	1.6 ± 1.0 ^a^	0.8	−67%
CABG with No Diabetes	2.7 ± 1.1	1.3 ± 0.5 ^a^	0.4	−52%
PCI with No Diabetes	3.7 ± 1.0	2.0 ± 0.9 ^a^	0.4	−46%
25–36 Sessions				
CABG with Diabetes	5.9 ± 0.8	4.4 ± 1.0 ^a^	0.4	−25%
PCI with Diabetes	5.1 ± 0.7	3.4 ± 0.6 ^a^	0.4	−33%
CABG with No Diabetes	5.0 ± 0.8	2.9 ± 0.6 ^a^	0.5	−42%
PCI with No Diabetes	3.4 ± 0.4	2.0 ± 0.3 ^a^	0.4	−41%

^a^ Denotes significant changes pre- to post-testing. Abbreviations: 12–24 Sessions = 12–24 sessions completed; 25–36 Sessions = 25–36 sessions completed; CABG = coronary artery bypass grafting; diabetes = type 1 and type 2 diabetes; PCI = percutaneous coronary intervention.

**Table 17:** Means ± SE for fasting blood glucose (mg/dL).

Condition	Pre	Post	Cohen’s d	% Change
12–24 Sessions				
CABG with Diabetes	141.3 ± 19.4	123.7 ± 10.8	0.3	−12%
PCI with Diabetes	222.4 ± 30.1	138.8 ± 16.8 ^a^	1.6	−38%
25–36 Sessions				
CABG with Diabetes	172.6 ± 64.1	141.2 ± 6.2	0.6	−18%
PCI with Diabetes	141.6 ± 8.7	136.3 ± 44.5	0.1	−4%

^a^ Denotes significant changes pre- to post-testing. Abbreviations: 12–24 Sessions = 12–24 sessions completed; 25–36 Sessions = 25–36 sessions completed; CABG = coronary artery bypass grafting; diabetes = type 1 and type 2 diabetes; mg/dL = milligrams per deciliter; PCI = percutaneous coronary intervention.

**Table 18:** Means ± SE for A1c.

Condition	Pre	Post	Cohen’s d	% Change
12–24 Sessions				
CABG with Diabetes	7.2 ± 0.6	6.2 ± 0.1 *	0.8	−14%
PCI with Diabetes	8.4 ± 0.6	7.8 ± 0.6 *	0.5	−7%
25–36 Sessions				
CABG with Diabetes	7.2 ± 0.2	6.8 ± 0.1 *	0.3	−6%
PCI with Diabetes	7.3 ± 0.2	6.7 ± 0.1 *	0.5	−8%

* Denotes significant changes pre- to post-testing. Abbreviations: 12–24 Sessions = 12–24 sessions completed; 25–36 Sessions = 25–36 sessions completed; CABG = coronary artery bypass grafting; diabetes = type 1 and type 2 diabetes; PCI = percutaneous coronary intervention.

**Table 19:** Percent change for means from pre- to post-test collapsed across all variables.

Condition	Pre	Post	% Change
Total Cholesterol	151.9 ± 3.3	129.6 ± 3.3	−14.7% *
Triglycerides	125.8 ± 4.9	104.9 ± 4.9	−16.6% *
High-Density Lipoprotein	41.8 ± 0.9	44.8 ± 0.9	7.1% *
Low-Density Lipoprotein	90.2 ± 2.8	66.5 ± 2.8	−26.3% *
Systolic Blood Pressure	119.8 ± 0.9	117.3 ± 0.9	−2.1%
Diastolic Blood Pressure	69.5 ± 0.7	66.3 ± 0.7	−4.5% *
Body Mass	85.3 ± 1.6	84.2 ± 1.6	−1.2%
Duke Activity Score Index	27.8 ± 1.1	42.7 ± 1.1	53.8% *
Patient Health Questionnaire-9	4.6 ± 0.3	2.6 ± 0.3	−44.1% *
Fasting Blood Glucose	163.0 ± 6.6	128.5 ± 6.6	−21.1%
A1c	7.3 ± 0.1	6.7 ± 0.1	−8.2%

* Denotes significant changes pre- to post-testing.

**Table 20:** Pearson’s correlations and *p* values for the number of rehabilitation sessions and pre- to post-test change for all variables.

Condition	Pearson’s *r*	*p*
Total Cholesterol	0.05	0.45
Triglycerides	0.09	0.18
High-Density Lipoprotein	−0.03	0.72
Low-Density Lipoprotein	0.05	0.51
Systolic Blood Pressure	0.00	0.98
Diastolic Blood Pressure	−0.03	0.64
Body Mass	0.08	0.29
Duke Activity Score Index	−0.03	0.69
Patient Health Questionnaire-9	0.11	0.13

**Table 21:** Pearson’s correlations and *p* values between the number of rehabilitation sessions and pre- to post-test change for the diabetic patients.

Condition	Pearson’s *r*	*p*
Fasting Blood Glucose	0.03	0.81
A1c	0.13	0.22

## Data Availability

The raw data supporting the conclusions of this article will be made available by the authors on request. Although anonymous and all patient identifiers have been removed, the hospital requested that the information not be shared publicly unless upon request.
